# The Role of Systemic Inflammation in the Pathogenesis of Spontaneous Intracranial Hemorrhage in the Presence or Absence of Effective Cerebral Blood Flow

**DOI:** 10.3390/jcm13154454

**Published:** 2024-07-30

**Authors:** Evgenii Gusev, Liliya Solomatina, Peter Bochkarev, Alevtina Zudova, Valeriy Chereshnev

**Affiliations:** 1 Institute of Immunology and Physiology Ural Branch of The Russian Academy of Sciences, 620078 Yekaterinburg, Russia; gusev36@mail.ru (E.G.); tina.zudova@mail.ru (A.Z.); v.chereshnev@mail.ru (V.C.); 2 Sverdlovsk Regional Clinical Hospital No. 1 (GAUZ SO “SOKB No. 1”), 620102 Yekaterinburg, Russia; bo4karevpeter@yandex.ru

**Keywords:** spontaneous intracerebral hemorrhage, coma, MODS, ineffective cerebral blood flow, systemic inflammation, cytokines, PCT, D-dimer, cortisol, NSE

## Abstract

**Background**: Spontaneous intracerebral hemorrhage (ICH) is one of the leading causes of mortality in intensive care units. The role of systemic hyperintense inflammation (SHI) in the pathogenesis of critical complications of ICH remains a poorly understood problem. There is a specific variant of severe ICH associated with increased intracranial pressure and occlusion of intracranial vessels, defined as ineffective cerebral blood flow (IECBF). **Methods**: To evaluate the role of SHI in the pathogenesis of severe (comatose) ICH in a dynamic comparison of patients with IECBF (n-26) and without IECBF (n-52). The SHI integral score criterion (SI scale) was used, including certain values of plasma concentrations of IL-6, IL-8, IL-10; TNF-α, PCT, cortisol, myoglobin, troponin I, D-dimer, and, additionally, SOFA scale values. Blood levels of ACTH and neuron-specific enolase (NSE) were also assessed. **Results**: Twenty-eight-day mortality in severe ICH reached 84.6% (without IECBF) and 96.2% (with IECBF). Clear signs of SHI were detected in 61.5%/87.8% (without IECBF) and 0.0%/8.7% (with IECBF) within 1–3/5–8 days from the onset of ICH manifestation. The lower probability of developing SHI in the IECBF group was associated with low blood NSE concentrations. **Conclusions**: The development of SHI in ICH is pathogenetically related to the permeability of the blood–brain barrier for tissue breakdown products and other neuroinflammatory factors.

## 1. Introduction

Spontaneous intracerebral hemorrhage (ICH), defined as nontraumatic bleeding into the brain parenchyma, is the second most common subtype of stroke, which accounts for 10–15% of all strokes with about half of them being fatal within a year [1,2]. Chronic arterial hypertension is a major risk factor for ICH [3], and the most common sites of hypertensive bleeding are the deep perforating arteries of the pontine, midbrain, thalamus, basal ganglia, and deep cerebellar nuclei [4]. Overall, ICH represents a potentially devastating subgroup of acute strokes with various manifestations, including primary intraparenchymal hematoma, intraventricular hemorrhage, and subarachnoid hemorrhage [5]. Preclinical and clinical trials have elucidated the underlying mechanisms of tissue damage from ICH, including the complex interplay between edema, inflammation, iron-induced damage, and oxidative stress [6,7]. The most common “anatomical” classification of ICH categorizes the brain regions directly damaged by ICH as lobar intracerebral hemorrhage (the junction of cortical gray and subcortical white matter) and deep hemispheric (basal ganglia, thalamus, internal capsule), brainstem (pons, midbrain, medulla), and cerebellum [8]. Examination of patients in coma commonly includes the Glasgow Coma Scale (GSC) score, which is very useful in patients with ICH [9].

When a stroke damages the brain, the immune system becomes overactive, which is very useful in patients with ICH [9], as this results in not only a local but also systemic inflammatory response and immune dysfunction and can significantly affect the prognosis of stroke [10]. Two major phenomena of peripheral immune dysregulation associated with stroke are systemic inflammation and post-stroke immunosuppression [11]. The characteristic systemic phenomena of ICH are leukocytosis, increased neutrophil/lymphocyte ratio, acute-phase liver response, increased blood levels of cytokines and various proteases, tissue degradation products, presence of systemic inflammatory response syndrome (SIRS) criteria, presence of signs of intravascular para-coagulation, and hypothalamic–pituitary–adrenal axis stress/distress [11,12,13].

The possible reasons for the development of systemic inflammation are pronounced neuroinflammation and impaired blood–brain barrier (BBB) function [12,14,15]. One of the most widely used methods to assess the condition the BBB and the degree of brain damage is the measurement of neuron-specific enolase (NSE) in blood [16,17]. Serum/plasma concentrations of NSE are significantly increased in stroke patients compared with controls and correlate with the severity of stroke symptoms [18,19].

Meanwhile, there is a special variant of severe complication in stroke associated with increased intracranial pressure and simultaneously decreased cerebral perfusion pressure that can lead to brain death due to the compression of intracranial vessels and impaired autoregulation of the tone of these vessels [20,21]. This condition is defined as inefficient cerebral blood flow (IECBF). It is associated with the consequences of cerebral edema, which increases intracranial pressure, leading to IECBF, up to complete blockade of cerebral perfusion and brain death [22]. Interestingly, in these cases, the metabolic processes between the brain and systemic blood flow may be disturbed, which allows for one to largely exclude generalizations of tissue breakdown products and local inflammation as trigger factors of critical systemic hyperinflammation (SHI). The usual method for determining the degree of IECBF is transcranial Doppler imaging to measure blood flow velocity in cerebral arteries, primarily in one of the middle cerebral arteries [23]. In this case, the so-called “zero flow” is a transcranial Doppler sign of complete cessation of cerebral blood flow, and it can be used as one of the proofs of brain death [24].

Considering the above, the aim of our study is to evaluate the role of SHI in the pathogenesis of severe (coma) ICH in a dynamic comparison of patients with and without the presence of IECBF.

## 2. Materials and Methods

### 2.1. Patient Characteristics

We studied three main groups of patients: C = control (blood donors), n = 89.3 ± 1.1 (Me ± SD) years, men = 50.6%; a group of patients with ICH without IECBF (Group 1); and a group of patients with ICH and IECBF (Group 2). ECBF was established by transcranial Doppler (TCD) imaging of the main arteries for oscillatory, multidirectional blood flow in systole/diastole as characteristic of IECBF. Thus, IECBF is verified when blood flow velocity (Vd) in the cerebral arteries is Vd < 0 in diastole and Vd > 0 in systole, in contrast to Vd = 0 (systole and diastole) at “zero blood flow”. This Doppler pattern in diastole is called reverberating blood flow (Vd < 0). In this case, both states of cerebral hemodynamics (in systole and diastole) do not support the necessary cerebral perfusion. Moreover, partial preservation of cranial nerve reflexes in this group of patients suggested partial preservation of brainstem function over the study period.

The patients were examined twice following admission to intensive care units: after 1–3 days (Groups 1A and 2A), and after 5–8 days (Groups 1B and 2B). The criteria for inclusion in Groups 1 and 2 were ICH, coma (GSC < 8 points), MOF (SOFA scale—Sequential Organ Failure Assessment), and need for artificial lung ventilation (ALV) in all patients. Thus, the general inclusion criteria for Groups 1 and 2 were the presence of severe ICH (coma), and for Group 2 only did we add the presence of IECBF.

The exclusion criteria were presence of “zero blood flow”, 48 h mortality, septicemic complications, stroke-related myocardial infarction, systemic autoimmune diseases, cancer, amyloidosis, HIV infection, viral hepatitis, tuberculosis, and other severe chronic infectious diseases. Also, none of the patients had surgery for ICH, hypothermia, hormone, and anti-cytokine therapies, or other anti-inflammatory medication. Additionally, fatal outcomes in the time interval between the two trials were excluded from group 1B, and patients who showed recovery of systemic cerebral blood flow in this time range were excluded from group 2B. Additional characterization of the patients is shown in Table 1. These data confirm the presence of significant changes in a number of homeostasis parameters in patients in both groups, including PON and coma in all patients and critical coma in most patients (GSC = 3 scores), as well as the establishment of brain death after the end of the study (after 8 days) in most patients of Group 2. The DIC syndrome was not diagnosed in patients of either group due to the absence of decreased plasma fibrinogen levels relative to the reference value (2 g/L) and prothrombin time deviations beyond the reference range established in the laboratory (11–18 min).

Data on other criteria for the DIC phenomenon (platelets, D-dimer) will be presented in Section 3. The majority of fatal outcomes in both groups were recorded 1–2 weeks after the end of this study. The fact that there were no patients in Group 2 exceeding the upper limit of normal NSE concentration in blood is an indirect sign of IECBF (Table 1).

Brain death (after the end of this study) in Group 2 patients was determined in case conferences of the medical institution’s experts, guided by Appendix No. 1 to the order of the Russian Ministry of Health dated 25 December 2014 No. 908n, which does not contradict the global agreement on BD/DNC accredited by 5 international federations and 27 medical professional communities around the world [25].

This study was conducted in accordance with the rules of the 1975 Declaration of Helsinki as revised in 2013. This study was approved by the ethical committees of the Institute of Immunology and Physiology UB RAS and Sverdlovsk Regional Clinical Hospital No. 1, and informed consent was obtained from the patients’ relatives for all subjects recruited for this study.

### 2.2. Measurement of Biomarkers

For the examinations, we used citrate-stabilized blood plasma pre-frozen at −20 °C. The levels of the SHI markers—interleukins (ILs) 6, 8, and 10; tumor necrosis factor alpha (TNF-α); procalcitonin (PCT); cortisol; ACTH; myoglobin; troponin I; NSE; and D-dimer—in blood plasma samples were analyzed by enzyme immunoassay on a Lazurit automated analyzer (Dynex, Zelienople, PA, USA).

### 2.3. Methods of SHI Verification and Assessment

This study uses integral criteria for SHI verification (SI scale), which we have previously tested in polytrauma, sepsis, obstetric massive blood loss, and various shock states [26,27,28]. For calculating the SHI scale, it is key to establish reactivity levels (RL 0–5 points), which reflect certain qualitative characteristics of the systemic inflammatory response (SIR) in critical conditions of varying etiology, including sepsis [29]. In addition, the SI scale verifies the following typical phenomena: disseminated intravascular coagulation (DIC), hypothalamic–pituitary–adrenal axis distress, systemic tissue alteration, and multi-organ failure (MOF). For the purposes of this study, we consider SHI from the perspective of general pathology as an independent type of general pathological process associated with life-critical microcirculatory disorders, which should be differentiated from SIR of canonical (classical) inflammation and systemic low-grade inflammation [30,31].

Based on the determination of five SIR indicators, we calculated the value of the integral reactivity level scale (RL scale) by summing and averaging the three highest values of the individual response levels of individual indicators (Table 2).

Interpretation of RL scale values [27,29]: RL-0 reflects SIR reference values; RL-1 excludes the presence of acute SHI; RL-2 and RL-3 require differentiation of SHI from hyperergic systemic phenomena of canonical inflammation; RL-4 and RL-5 characterize the cytokine storm phenomenon; and RL-5 verifies SHI regardless of the presence of other SHI criteria determined using a more integral SI scale (Table 3).

SI scale interpretation [26,27]: (1) ≥5 points with RL ≥ 2 verifies SHI; (2) 3–4 points with RL ≥ 1 is a marginal state, namely, pre-SHI; (3) ≤2 points of the SHI scale with RL-1 or RL-2 confirms the presence of SIR without association with SHI; (4) ≤2 points with RL-0 does not confirm the presence of SIR and SHI.

### 2.4. Statistical Analysis

Statistical analysis was performed using the Statistica 12.0 program (Stat Soft, Inc., Tulsa, OK, USA). Descriptive statistics are presented according to their main characteristics: Me is median, SD is standard deviation, and 25% and 75% are quartiles. Kolmogorov–Smirnov and Shapiro–Wilk tests were used to test the hypothesis if the sample distribution was non-normal. Comparisons between groups were performed using the Mann–Whitney test, the paired Wilcoxon test was applied to related (dependent) groups of patients, and Pearson’s chi-squared test (χ^2^) was used for categorical variables. Nonparametric Spearman rank correlation coefficients were used to assess the strength and direction of association between variables. All results were considered statistically significant if *p* < 0.05.

## 3. Results

All differences in the data presented in this section were interpreted as significant at *p* < 0.05. The condition of the patients in both groups was characterized as stably severe, with changes in a number of homeostasis parameters (Table 4). Comparison of groups 1A/2A and 1B/2B shows that, the follow-up periods being comparable, changes in Groups 2 are more pronounced than in Groups 1.

**Figure 1:**
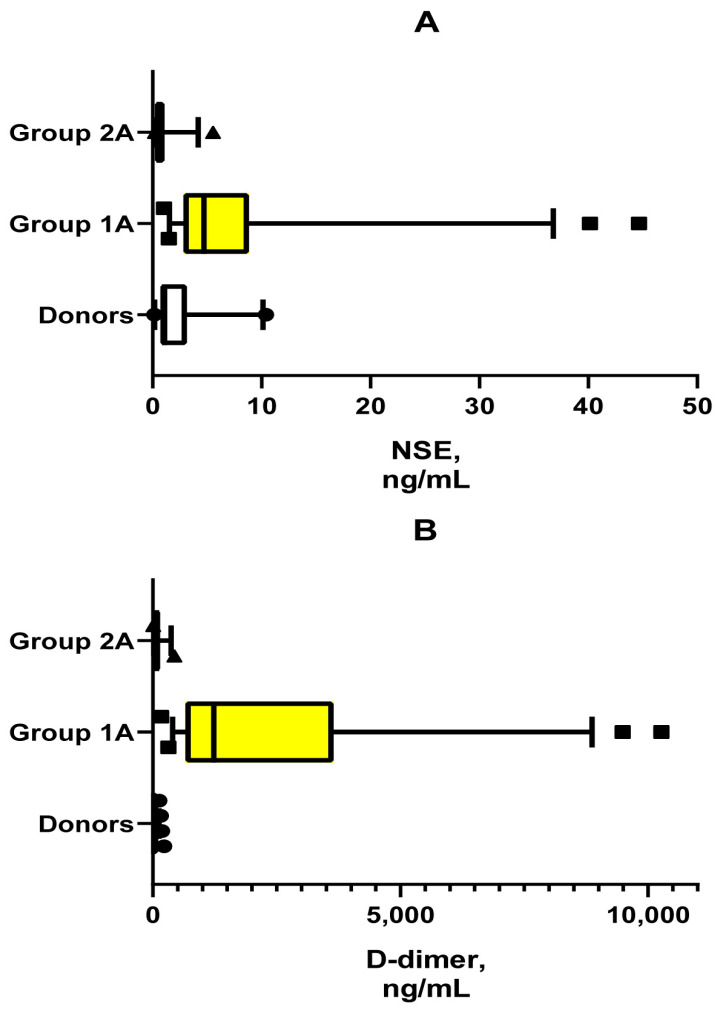
Ratio of plasma concentrations of NSE (**A**) and D-dimers (**B**). Note. The graphs show data from 5th to 95th percentiles. The shapes indicate outliers. The median of the data groups is also shown. The reliability of statistically significant differences in data is presented in Table 5.

Conversely, the quantitative increase of individual SHI indices relative to controls is significantly different in most cases in all four groups of ICH patients (Table 5). Meanwhile, these changes are more significant in Groups 1 than in Groups 2. At the same time, there is a distinct tendency for many of these indices to be increased at re-examination, particularly between groups 1A/1B and 2A/2B (Table 5). Alongside the above, we revealed two regularities: (1) activation of the hypothalamic–pituitary–adrenal axis function (ACTH, cortisol) in both Groups 1A and 2A, and (2) multidirectional changes in NSE blood levels in Groups 1A (increase) and 2A (decrease) relative to the control. Figure 1 contrasts the multidirectionality in these changes. In addition, Figure 1 shows a pronounced elevation of D-dimer levels in Group 1A compared with both control and Group 2A.

Correlation analysis (Table 6) using pooled data from groups 1A + 2A shows a positive correlation (criterion: R > 0.4 for *p* < 0.05) between NSE and the majority of the SHI scores, but a negative correlation with the SOFA scores (R < −0.4 for *p* < 0.05). This study demonstrates that if one does not differentiate ICH into groups with and without IECBF, the result may be paradoxical, lacking association between the severity of the patients’ condition (SOFA scale) and the extent of damage to NSE integrity.

Table 7 presents data for the study groups on the percentage distribution of RL values (0–5), SHI phenomena, as well as the presence of pre-SHI and SHI obtained using the SI scale.

In Group 2A, there are no signs of SIR (100% RL-0), as the changes detected in individual SIR parameters are in most cases within the reference intervals. Neither did we verify other SHI phenomena in this group apart from MOF (100%). Overall, this does not verify the presence of pre-SHI and SHI in Group 2A patients. In Group 2B, the expression of individual SHI phenomena is noted in only some patients, allowing for SHI to be verified in 8.7% of patients.

A completely different picture is observed in the groups of patients without IECBF, including higher RL values, expression of private SHI phenomena, and, most importantly, the presence of pre-SHI/SHI: 33.3/60.9% in Group 1A and 8.2/87.8% in Group 1B. Moreover, the differences between Groups 1A/1B in terms of SHI expression are significant (*p* < 0.05). Signs of a cytokine storm (RL–4–5) were observed only in 7.9% of patients in Group 1A and 18.4% of patients in Group 1B.

Thus, the results of this study demonstrate a significant and ever-increasing importance of systemic hyperinflammation in the pathogenesis of ICH. By contrast, in the groups with IECBF (2A and 2B), there were no manifestations of this pathologic process, or they were present in just a small number of patients (8.7%) in group 2B.

## 4. Discussion

Currently, there is no doubt concerning the role of cytokines and other neuroinflammatory factors as secondary inducers of brain tissue damage during stroke [32,33,34]. Cytokines and other systemic inflammatory factors are also known to be inducers of peripheral tissue damage, including in ICH [11,13,35]. Meanwhile, the question of the differential role of the generalization of neuroinflammation products in the development of SHI on the one hand and critical impairment of brain function (coma) under conditions of artificial life support is still open. To address this question, we compared the probability of SHI development in two groups of patients comparable in ICH severity, namely, with and without IECBF. In the first case, the generalization of tissue damage and neuroinflammation factors will be significantly delayed, which is confirmed by the low level of NSE in the blood.

**Figure 2:**
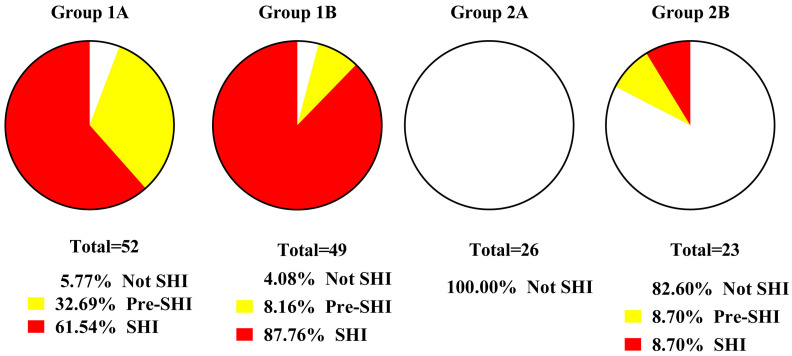
The severity of systemic inflammation and presystemic inflammation (%) in the studied groups. Note: SHI—systemic hyperinflammation; Pre-SHI—presystemic inflammation.

Despite the critical condition of all patients with ICH, the involvement of SHI in the pathogenesis of this disease was found in most patients without IECBF and only in some patients with IECBF in Group 2B (Figure 2). The main reason for this discrepancy seems to be related to the lower likelihood of generalization of neuroinflammatory products in patients with the presence of IECBF. Indirectly supporting this statement are lower NSE levels in patients with IECBF not only in comparison with Group 1A, but also against controls. Meanwhile, 21.8% of the patients in Group 1A met criteria for blood–brain barrier integrity disruption (NSE > 17.6 ng/mL). Conversely, moderate (borderline) changes in SIR and D-dimer blood levels in the majority of IECBF patients may be related not only to the reaction to brain damage, but also to the presence of chronic systemic low-grade inflammation. This type of inflammation is characterized by endotheliosis, thrombophilia, insulin resistance, marginal SIR levels, and the presence of such clinical risk factors for ICH as neurodegeneration, hypertension, and atherosclerosis [36].

However, these changes cannot characterize SHI as a critical complication of acute infectious and aseptic diseases. In general, SHI in ICH has all the characteristic features of this type of pathological process, but with some specifics in its dynamics. Thus, for sepsis and polytrauma in the intensive care context, the probability of SHI verification is highest in the first three days of critical condition, being characterized by a greater expression of the cytokine storm phase than in ICH [27]. SHI/ICH shows an even greater distinction from the dynamics of superacute SHI associated with the lightning form of DIC syndrome, such as in amniotic embolisms. In the latter case, a refractory distributive shock sets in already in the first hours of the critical state, with pronounced manifestations of a cytokine storm developing by hour 5–8 (RL-5) [28].

By contrast, in SHI/ICH, the DIC phenomenon (high D-dimer levels) does not fully meet the criteria for the DIC syndrome, and the SHI process develops more slowly than other variants of acute SHI [37,38]. The relatively lower probability of SHI development in patients with IECBF probably makes these patients more preferable candidates as transplant organ donors, once brain death has been verified in them. This is due to the fact that SHI is a pathogenetic factor of secondary systemic organ damage capable of worsening the condition of various donor organs. The latter phenomenon has been confirmed in the present study by determining the levels of myoglobin and myocardium-specific troponin I in blood plasma.

This study has several limitations. First, the design of this study does not allow for accurate determination of the possible association between SHI and death, especially in the group with IECBF, because biological death following brain death establishment is determined by discontinuation of intensive care treatment; where brain death was not established, deaths were recorded in this category of patients beyond the study deadline. The latter is true for the majority of fatal outcomes in Group 1 (without IECBF). Second, we did not investigate all variants of severe SHI. Third, we did not explore the effect of anti-inflammatory therapy on the prevention and management of SHI/ICH. Fourth, our criteria proposed for the verification and pathogenetic evaluation of SHI are just one possible method to solve this problem, and the degree of plausibility of this particular method of SHI evaluation needs to be ascertained. Meanwhile, our proposed method and methodological approach currently have no alternative options for the assessment of SHI as a general pathologic process rather than a private clinical phenomenon.

In our opinion, the development and implementation of new protocols for pathogenetic therapy of severe ICH aimed at SHI management will be a promising way to solve this problem. It is also advisable to monitor SHI phenomena in potential organ donors, given that SHI is a powerful factor of secondary systemic damage that disrupts the integrity of all vital organs.

## 5. Conclusions

The findings of this study and their analysis and systematization allow for us to draw the following main conclusions:

1. Severe ICH (coma, MOD, ALV) both without and with IECBF are extremely critical conditions with a high percentage of 28-day mortality at 80.8% (without IECBF) and 96.2% (with IECBF).
2. Verification of systemic hyperinflammation using integral criteria in the ranges of days 1–3/5–8 from the onset of critical condition show that in Group 1 (without IECBF), the presence of SHI is detected in 60.9/78.8%, while in Group 2 (with IECBF) in just 0.0/8.7%.
3. The low probability of SHI development in the group with IECBF is pathogenetically associated with low blood concentrations of NSE, a marker of blood–brain barrier permeability for brain tissue damage products and other neuroinflammatory factors.

## Figures and Tables

**Table 1:** General characteristics of the patients.

Evaluation Parameters	Group 1 (without IECBF)	Group 2 (with IECBF)
n, 1–3/5–8 days	52/49	26/23
Age, years, Me ± SD (Group 1A and 2A)	54.0 ± 11.5	50.5 ± 10.8
Paul f/m, %, (Group 1A and 2A)	50/50	36/64
Intracerebral hemorrhage intraventricular, %	21.1 *	42.3 *
Multiple organ failure (MOF), %	100	100
Artificial lung ventilation (ALV), %	100	100
Critical coma (GSC = 3 scores), %	82.7	76.9
28-day mortality, %	84.6	96.2
Criteria DIC syndrome, %	17.3	0
Treatment with vasopressors, %	23.1 *	80.8 *
NSE > 17.6 ng/mL ^1^, %	0	21.8 *
28 daily brain death ^2^ (group 2B, n-23), %	0	87.0 *
Organ donors ^3^ (group 2B, n-23), %	0	21.8 *

Note. *—significant differences (*p* < 0.05) as %. ^1^—reference NSE intervals 0.0–17.6 mg/mL (Roche Diagnostics GmbH); ^2^—fatal outcome stated as a result of brain death verification; ^3^—in organ transplant; GSC—Glasgow Coma Scale; NSE—neuron-specific enolase.

**Table 2:** Calculation of integral RL scale.

Factors	Norm Values	Values of Individual Reactivity Levels						
		0	1	2	3	4	5	6
IL-6, pg/mL	≤5	≤5	≤10	≤40	≤200	≤1000	>1000	no
IL-10, pg/mL	≤10	≤10	no	≤20	≤50	≤200	≤1000	>1000
IL-8, pg/mL	≤10	≤10	≤25	≤100	≤500	≤2500	>250	no
TNF-α, pg/mL	≤8	≤8	≤16	≤40	≤160	≤800	>800	no
PCT, ng/mL	≤0.1	≤0.1	≤0.25	≤1	≤10	≤100	>100	no

Note. Calculation of integral RL scale (0–5 points): RL-0 (0–1), RL-1 (2–4), RL-2 (5–7), RL-3 (8–10), RL-4 (11–13), RL-5 (14–16). Numerals in brackets represent ranges of sums of three highest values of individual SIR levels. IL—interleukin; TNF-α—transforming growth factor alpha; PCT—procalcitonin.

**Table 3:** Methodology for calculating the integral scale of acute SI.

Phenomenon of SHI	Criterion	Points	Note
Systemic inflammatory reaction	RL scale (0–5 points)	2–5	RL 0–1 rules out the presence of an acute
Disseminated intravascular coagulation	D-dimer > 500 ng/mL	1	or the presence of criteria for DIC syndrome
Distress of the hypothalamic–pituitary–adrenal axis	Cortisol ^1^ >1380 or <100 nmol/L	1	In the absence of a criterion, but with glucocorticoid treatment, 1 point is added
Systemic tissue alteration	Troponin I ≥ 0.2 ng/mL and/or myoglobin ≥ 200 ng/mL	1	Troponin I is not taken into account in myocardial infarction
Multiple organ failure	Scale SOFA	1	or other criteria for multiple organ dysfunction syndrome

Note. Each phenomenon is assigned a certain score by the SHI scale, and then the scores are summed up. ^1^—the reference range of cortisol levels in blood plasma 138–690 nmol/L.

**Table 4:** Individual homeostasis indicators in the studied patient groups.

Indicators	1A	1B	2A	2B
Heart rate (N 60–90)	85.0 ± 21.4	89.0 ± 17.1	93.0 ± 35.7	100.0 ± 31.0
Average blood pressure	101.3 ± 23.2 *	99.7 ± 20.8 *	76.7 ± 16.6	73.7 ± 11.5
Central venous pressure (N 8–12 mmHg)	12.0 ± 2.1	12.0 ± 2.9	12.0 ± 3.7	12.0 ± 3.4
Bilirubin (N < 20 μmol/L)	33.0 ± 11.7	33.5 ± 11.9	36.0 ± 11.3	36.0 ± 12.7
Creatinine (N < 110 mmol/L)	93.0 ± 41.2 *	151.5 ± 63.3 *	67.0 ± 39.7	67.0 ± 33.9
Urea (N 1.7–8.3 mmol/L)	7.7 ± 4.6	11.3 ± 4.8 *	7.8 ± 4.3	7.6 ± 4.0
Platelets (N 180–320 × 10^3^/mm^3^)	201.0 ± 95.1	208.5 ± 94.9	213.0 ± 140.1	134.0 ± 94.5
Glucose mmol/L ^1^	10.5 ± 4.4	12.6 ± 3.6	11.6 ± 4.5	11.7 ± 4.8
ALT (N < 40 units)	49.0 ± 31.9 *	92.5 ± 75.5 *	31.0 ± 42.7	25.0 ± 47.7
AST (N < 40 units)	65.0 ± 56.1 *	88.0 ± 66.2 *	27.0 ± 26.4	26.0 ± 25.6
Na^+^ (N 136–145 mmol/L)	143.0 ± 10.5 *	146.0 ± 11.2 *	154.0 ± 10.1	156.0 ± 9.1
K^+^ (N 3.5–5.2 mmol/L)	4.2 ± 0.8	3.7 ± 0.9	4.6 ± 0.8	4.5 ± 1.2
Cl^−^ (N 98—113 mmol/L)	105.0 ± 3.8	105.0 ± 4.3	104.0 ± 5.1	104.5 ± 4.5
SpO_2_/FiO_2_	291.3 ± 75.7	291.9 ± 62.5	310.0 ± 60.7	307.5 ± 85.1

Note. Data are presented as Me ± SD; *—significant differences (*p* < 0.05) between groups: 1A/2A and 1B/2B. N—normal (reference intervals). ^1^—N of glucose when measured in the morning before eating 3.3–5.5 mmol/L. ALT—alanine aminotransferase; AST—aspartate aminotransferase.

**Table 5:** Data of intergroup differences of the studied indicators.

Indicators	Control	Group 1A	Group 1B	Group 2A	Group 2B
SOFA, scores	0 [1A, 1B, 2A, 2B]	7.00 (6.00–8.00) [C, 1B, 2A, 2B]	9.00 (8.00–10.00) [C, 1A, 2A, 2B]	10.00 (9.00–11.00) [C, 1A, 1B, 2B]	11.00 (10.00–12.00) [C, 1A, 1B, 2A]
Myoglobin, ng/mL	7.74 (5.61–13.28) [1A, 1B, 2A, 2B]	79.40 (45.69–173.6) [C, 2A, 2B]	123.90 (50.58–230.73) [C, 2A, 2B]	16.15 (13.88–19.15) [C, 1A, 1B]	18.05 (14.50–26.75) [C, 1A, 1B]
Troponin I, ng/mL	0.00 (0.00–0.00) [1A, 1B]	0.05 (0.02–0.349) [C, 2B, 2A]	0.07 (0.03–0.31) [C, 2A, 2B]	0.00 (0.00–0.00) [1A, 1B]	0.00 (0.00–0.00) [1A, 1B]
D-dimer, ng/mL	11.5 (4.9–30.3) [1A, 1B, 2A, 2B]	1228.4 (654.0–3654.0) [C, 1B, 2A, 2B]	2517.6 (1294.8–4848.0) [C, 1A, 2A, 2B]	75.0 (42.5–91.6) [C, 1A, 1B]	105.0 (31.5–216.3) [C, 1A, 1B]
IL-6, pg/mL	0.80 (0.45–1.36) [1A, 1B, 2A, 2B]	78.35 (42.80–223.10) [C, 1B, 2A, 2B]	185.32 (77.82–317.70) [C, 1A, 2A, 2B]	1.40 (1.20–1.50) [C, 2B, 1A, 1B]	6.25 (2.20–12.28) [C, 1A, 1B, 2A]
IL-10, pg/mL	0.57 (0.00–21.95) [1A, 1B, 2A]	10.90 (5.37–18.02) [C, 2A, 2B]	14.46 (4.90–25.10) [C, 2A, 2B]	1.75 (0.63–4.30) [C, 1A, 1B]	0.70 (0.23–4.68) [1A, 1B]
IL-8, pg/mL	1.72 (1.35–2.38) [1A, 1B, 2A, 2B]	16.40 (5.50–48.45) [C, 1B, 2A]	50.10 (14.70–127.46) [C, 1A, 2A, 2B]	3.00 (2.90–3.18) [C, 1A, 1B, 2B]	6.70 (3.53–37.28) [C, 1B, 2A]
TNF-α, pg/mL	0.00 (0.00–0.36) [1A, 1B, 2A, 2B]	1.36 (0.20–4.00) [C, 1B]	7.62 (1.05–20.40) [C, 1A, 2A, 2B]	1.34 (0.93–1.54) [C, 1B, 2B]	1.54 (1.27–1.87) [C, 1B, 2A]
PCT, ng/mL	0.03 (0.02–0.03) [1A, 1B, 2A, 2B]	0.89 (0.40–2.96) [C, 2B, 2A]	1.26 (0.48–3.84) [C, 2A, 2B]	0.10 (0.10–0.11) [C, 1A, 1B]	0.11 (0.10–0.16) [C, 1A, 1B]
Cortisol, nmol/L	358.3 (278–450) [1A, 1B, 2A, 2B]	898.5 (467–1387) [C]	1006.8 (560.0–1475.0) [C, 2A]	564.5 (535–575) [C, 1B]	565.5 (543–871) [C]
ACTH, pg/mL	2.40 (1.36–3.64) [1A, 1B]	4.00 (2.70–9.20) [C]	No	6.30 (5.82–6.55) [C]	No
NSE, ng/mL	0.99 (0.71–3.83) [1A, 1B]	4.71 (2.85–8.79) [C, 1B]	No	0.60 (0.60–0.70) [C, 1A]	No

**Table 6:** Comparison of parameters with neuron-specific enolase according to the Spearman test.

Indicators	Spearman—R	*p*-Value
SOFA	−0.705673	0.000000
Myoglobin	0.649737	0.000000
D-dimer	0.774284	0.000000
IL-6	0.730473	0.000000
IL-10	0.440968	0.000167
IL-8	0.591767	0.000000
PCT	0.655436	0.000000
TNF-α	0.099814	0.418025
Cortisol	0.331982	0.005679
Troponin I	0.728685	0.000000
RL	0.767600	0.000000
Scale SI	0.799648	0.000000

Note. Data with significant differences (*p* < 0.05) relative to the indicated value are presented in square brackets. IL—interleukin; TNF-α—tumor necrosis factor alpha; PCT—procalcitonin; RL—reactivity level; SI—systemic inflammation.

**Table 7:** Frequency analysis (in %) of SHI criteria in the studied patient groups.

Indicators	Group 1A	Group 1B	Group 2A	Group 2B
RL-0	3.9 [2A, 2B]	4.1 [2A, 2B]	100 [1A, 1B]	68.2 [1A, 1B]
RL-1	15.7 [2A, 1B]	2 [1A]	0 [1A]	9.1 [No]
RL-2	31.4 [2A]	22.4 [2A]	0 [1A, 1B]	18.2 [No]
RL-3	41.2 [2A, 2B]	53.1 [2A, 2B]	0 [1A, 1B]	4.5 [1A, 1B]
RL-4	5.9 [No]	18.4 [2A, 2B]	0 [2B]	0 [2B]
RL-5	2.0 [No]	0 [No]	0 [No]	0 [No]
Troponin I > 0.2 ng/mL	33.3 [2A, 2B]	40.8 [2A, 2B]	0 [1A, 1B]	4.3 [1A, 1B]
Myoglobin > 200 ng/mL	19.6 [2A, 2B]	34.7 [2A, 2B]	0 [1A, 1B]	0 [1A, 1B]
Tissue alteration ^1^	43.1 [2A, 2B]	59.3 [2A, 2B]	0 [1A, 1B]	4.3 [1A, 1B]
Cortisol > 1380 or < 100 nmol/L	25.5 [2A]	40.1 [2A, 2B]	0 [1A, 1B]	8.7 [1B]
D-dimer > 500 ng/mL	92.2 [2A, 2B]	95.9 [2A, 2B]	0 [1A, 1B]	8.7 [1A, 1B]
Multiple organ failure (SOFA)	100 [No]	100 [No]	100 [No]	100 [No]
Pre-SHI	32.7 [2A, 2B, 1B]	8.2 [1A]	0 [1A]	8.7 [1A]
Scale SHI	61.5 [2A, 2B, 1B]	87.8 [2A, 2B, 1A]	0 [1A, 1B]	8.7 [1A, 1B]

Note. Groups with significant differences by χ^2^ criterion (*p* < 0.05) are indicated in square brackets. ^1^—integral index: troponin I ≥ 0.2 ng/mL and/or myoglobin ≥200 ng/mL; RL—reactivity level; SIR—systemic inflammatory response; SHI—systemic hyperinflammation. Control data: RL-0 (98.9%), RL-1 (1.1%), other SHI criteria—0%.

## Data Availability

The datasets analyzed in the current study are available from the corresponding author upon reasonable request because they contain information on the gender, age, and diagnosis of the patients.
